# Unsuitable Decisions and Persistent Infection: The Reality of Farmer and Veterinarian Mastitis Treatment Decisions in Denmark

**DOI:** 10.3390/antibiotics15060570

**Published:** 2026-06-03

**Authors:** Luc Durel, Troels Volhøj, Fabienne Benoît, Michael Farre

**Affiliations:** 1 VIRBAC S.A., 06510 Carros, France; 2 VIRBAC Danmark A/S, 6000 Kolding, Denmark; troels.volhoj@virbac.dk; 3 LABEO Manche, 50000 Saint-Lô, France; 4 SEGES P/S, 8200 Kolding, Denmark; mifa@seges.dk

**Keywords:** clinical mastitis, selective treatment, diagnostic, decision, accuracy

## Abstract

**Background/Objectives**: Reducing antibiotic use in the dairy sector is a critical objective. This study aimed to assess whether Danish farmers were able to achieve this objective appropriately without affecting animal health. **Methods**: Participants chose from three options—antibiotics (ABs), no treatment (NO), and non-steroidal anti-inflammatory drugs (NSAIDs)—and their decisions were compared against a laboratory-defined ‘gold standard’. **Results**: The results indicated that farmers’ professional judgment was unreliable; they frequently misallocated antibiotic treatments, often failing to treat cows that needed them while treating those that did not. Overall, producers administered significantly fewer antibiotics than laboratory results suggested were necessary. Although veterinarians achieved better results via in-clinic bacteriological examinations, their ability to identify specific bacterial species was insufficient. Consequently, cows remain at a significant risk of persistent infection following a clinical episode, regardless of the farmer’s chosen treatment. **Conclusions**: The study concludes that significant progress needs to be made both on farms and in veterinary clinics to ensure that the use of antibiotics is as little as possible, but as much as necessary. Until such reliability improves, unsuitable treatment will continue to compromise animal health.

## 1. Introduction

Clinical mastitis (CM) is among the most common and damaging diseases in dairy cows. It is an infection of the mammary gland that can develop rapidly and become severe. By the late 1960s, the local or systemic administration of antibiotics—alongside the culling of infected animals—became the primary method for eliminating intramammary infections (IMI) during lactation and at dry-off [1,2]. The *National Institute for Research in Dairying* (NIRD) in Great Britain standardized the so-called “5-point plan,” establishing a foundational operational principle for the dairy industry: detect CM early and treat every case effectively [3,4]. Technical extension materials from that era were prescriptive; for instance, a 1970 *Milk Marketing Board of England & Wales* pamphlet advised producers to *consult* [their] *vet about the best antibiotic to use on* [their] *farm*. *Treat immediately any clot or udder symptom you see, and give the full course of treatment your vet recommends* (leaflet depicted in [4]). While this framework has been highly successful in reducing infection rates, it has resulted in a significant concentration of antimicrobial use within the dairy industry. Currently, although intramammary drugs constitute approximately 1% of total veterinary antimicrobial consumption in Europe [5], IMI treatments account for 70% of the volume used within the dairy industry, one-third of which is dedicated specifically to CM [6].

Despite their efficacy, these historical guidelines encompass two critical facets with unaddressed underlying premises. First, mastitis diagnosis begins with visual observation. The role of the milker in this process is undisputedly critical [7]. Consequently, since CM cases are detected by farm staff, most treatments of non-severe CM are performed by farm workers without direct veterinary supervision [8], and only a few of them routinely consult with their veterinarians regarding milk quality [9], a trend that is likely consistent across North America and Europe. While farmers are prepared to treat any clot or udder symptom immediately, the fact that the therapeutic decision-making power rests almost exclusively with them is often overlooked. Second, the veterinarian’s contribution can be summarized in three words: “consult your vet.” It was traditionally assumed that the veterinary surgeon—by virtue of education, professional status, or role as a primary authoritative source of knowledge, a “walking encyclopedia” [10]—held the definitive rules for antibiotic selection. This judicious use remained a “blind spot” during the era of the 5-point plan.

It is acknowledged that mastitis diagnosis is often a challenge for veterinarians. Bacteriological culture (BC) is one of the most frequently used diagnostic tools to resolve mastitis problems [7]. Identification of mastitis-causing organisms on individual farms has long been recognized as a key component of prevention planning [9]. A more recent development has been the use of pathogen identification to inform individual cow treatment protocols for clinical mastitis during lactation, with the potential to further reduce antimicrobial use [11]. For CM diagnosis, it is important that BC results are available as soon as possible to optimize treatment results, save costs, and prevent the ineffective use of antibiotics [7].

In Denmark, farmers are permitted to treat clinical mastitis independently. However, only benzyl penicillin can be used, and every application of an antibiotic must be accompanied by a milk sample that is transferred to a laboratory as soon as possible after the clinical outbreak. Depending on their experience with the disease, farmers can also opt for supportive care alone—primarily non-steroidal anti-inflammatory drugs (NSAIDs)—or forgo treatment entirely. This constitutes the primary level of clinical decision-making, which must be documented via an official reporting platform. Subsequently, milk samples are transported to a laboratory for milk culture. The local laboratory is typically the veterinary clinic in charge of the sanitary supervision of the farm. The in-clinic laboratory identifies the causative pathogens of the IMIs, providing data that could theoretically support a secondary level of culture-based treatment decisions. In the present study, a second milk sample was submitted to a reference laboratory for definitive bacterial identification. However, neither the in-clinic nor the reference laboratory results were available in a timeframe sufficient to support the farmer’s initial therapeutic choices.

The objective of this study was to compare actual farmer-led treatment decisions with potential decisions that would have been made by veterinarians or informed by laboratory diagnostics. Furthermore, the study evaluated the subsequent consequences of these varying decision-making paths on the health outcomes of affected cows and their long-term retention within the herd.

## 2. Results

### 2.1. Study Population

This study was a prospective observational field study conducted across eleven dairy farms and four veterinary clinics, in which quarter milk samples and associated treatment decisions were recorded over a three-month period under routine management conditions, where the study unit was quarter-level samples.

### 2.2. Diagnostic of Intramammary Infections

#### 2.2.1. Causative Bacterial Species Identified by the Reference Laboratory

In this study, laboratory results showed that IMI epidemiology was dominated by Streptococci (*Streptococcus uberis*, *Str. dysgalactiae*) in 41.9% of cases and by Gram-negative bacteria (*Escherichia coli*, *Klebsiella pneumoniae*) in 16.1% of cases. In total, (n = 22) distinct species were identified (Supplementary Material Table S1). To make further comparisons possible, results were split into nine categories (negative culture, NEG, contaminated culture, CONT, mixed culture, MIX, *Staphylococcus aureus*, SAU, non-aureus staphylococci and mammaliicocci, NASM, *Streptococcus*. *uberis*, SUB, other G-positive bacteria, OGP, coliforms, COL, and other Gram-negative bacteria, OGN) (Supplementary Material Figure S1).

#### 2.2.2. Identification of Causative Bacteria in Veterinary Clinics

Veterinary results paralleled those of the laboratory. When results were grouped in four broad culture categories (MIX/CONT, G+, G−, NEG) that make sense in the perspective of treatment selection, in-clinic results converged with laboratory findings, and it was not possible to detect differences between the two series (χ^2^ = 0.934, *p* = 0.817) (Table 1).

However, a detailed comparison revealed significant discrepancies between the in-clinic milk culture diagnostic and the laboratory diagnostic; species identification matched in only 52.1% of cases (Supplementary Material Table S1). Veterinarians frequently misidentified contaminated samples and failed to distinguish species-*specific* NASM, instead classifying them all as coagulase-negative staphylococci (CNS), indistinctively. Critical species—including the predominant *Str. uberis*, as well as *S. aureus* and *Str. agalactiae*—were often misidentified, though coliforms were identified with reasonable accuracy. Practitioners also reported unusual species not confirmed by the laboratory (e.g., *Candida*, *Micrococcus*). These errors highlight shortcomings in both sensitivity (Se) and specificity (Sp): while veterinarians correctly identified (Se) 57.1% of *Str. uberis*, 42.9% of cultures identified as *Str. uberis* were false positives (1 − Sp).

### 2.3. Treatment Application

#### 2.3.1. Treatment Incidence

Based on the cattle database records, the annual incidence rate of CM treatment is estimated to be 19 cases per 100 cow-years (n = 19). At the very last moment, treatment selection was the farm staff’s decision, only guided by the specific mastitis care SOP of the farm. In such a situation, 58.1% of CM (n = 160) were reported with AB treatment, and 15.6 and 26.3% were treated with NS or not treated at all (NO), respectively.

#### 2.3.2. Farm Staff Treatment Decision

It is possible to link the treatment decisions taken on the farm to the broad categories of germs identified in the milk by the laboratory (Table 2). With respect to the Gram staining, only 54.7 to 62.5% of Gram-positive-related IMI were treated with ABs, whereas 50.0 to 60.0% of Gram-negative-related IMIs received ABs, and one negative culture out of two was also treated with ABs.

#### 2.3.3. Relative Risk of Antibiotic Treatment

Compared to the farm-specific treatment protocol, the optimal treatment strategy based on laboratory results shows that producers solely treat a proportion of the identified CM with ABs. In our dataset, 134/174 CM might have benefited from an AB treatment because of the evidence-based nature of the infection; only 93/160 were actually treated. The relative risk (RR) of antibiotic treatment ranged from 0.423 to 1.429 across the 11 farms, with an average RR of 0.755 (CI_95%_ [0.647; 0.881], *p* < 0.001). Dairy producers treated significantly less cases with ABs than probably needed.

If veterinarians had to select one of the three treatments based on their own results, and according to the guidelines reported in Table 1, it is estimated that RR = 0.911 (CI_95%_ [0.797; 1.042], *p* = 0.165). Regarding the use of ABs, the veterinarian’s decision was likely to be appropriate.

#### 2.3.4. Treatment Adequacy

Overall, farm staff decision for a treatment option (AB, NO or NS) was in line (accuracy) with potential recommendations deduced from laboratory results in 78/160 (48.8%) of cases only (Table 3). Bringing results together showed that 54/123 of the cases that would likely benefit from an AB treatment were not treated; on the contrary, 24/37 animals were given unnecessary AB treatment. The performance of the farm staff in selecting AB treatment when it mattered was 0.561 in sensitivity (Se) and 0.351 in specificity (Sp). The negative predictive value (NPV), or the probability that a cow did not actually need AB while the farm staff decided to give NS or NO was as low as 0.194. Conversely, the positive predictive value (PPV) was 0.742.

On the veterinary side, practitioners’ decisions were potentially a good fit with laboratory results in 120/144 cases (83.3%), with only 13/108 animals left without AB treatment and 6/36 receiving AB treatment when it was not needed (Table 4). The veterinary test provided information about the necessity of AB treatment with a performance estimated at Se = 0.880, Sp = 0.833, NPV = 0.698 and PPV = 0.941.

### 2.4. Udder Health Outcomes

#### 2.4.1. Udder Health Status and Evolution

Treatment outcomes are not reported by producers. However, it can be estimated in assessing udder health status before the clinical episode and after the CM episode. Based on pre-CM cow composite somatic cell counts from the penultimate and last test days (td − 1, td − 2), and with the available information, 45/146 were deemed inflamed (INF) before the clinical event. After CM and treatment (AB, NO, or NS), the proportion of INF and healthy (HTY) animals was similar—74/146 vs. 72/146, respectively. The INF to HTY ratio before and after CM differed significantly (χ^2^ = 11.928, *p* = 0.001). For a cow with CM, the odds of being inflamed after a CM episode is OR = 2.307 times higher than before (CI_95%_ [1.430; 3.721]; *p* < 0.001).

#### 2.4.2. Treatment Outcomes

The evolution of the udder health status with regard to the treatment applied is reported in Table 5. The clinical episode and its treatment, if any, was a failure (unsuccessful) in 50.7% of cases, and it was inconsequential or a success in 49.3%. Although AB and NS treatments performed slightly better than NO, no significant difference was detected among treatments regarding a shift in the udder health status (one-way ANOVA, F = 2.742, *p* = 0.143).

### 2.5. Retention Within the Herd

**Figure 1:**
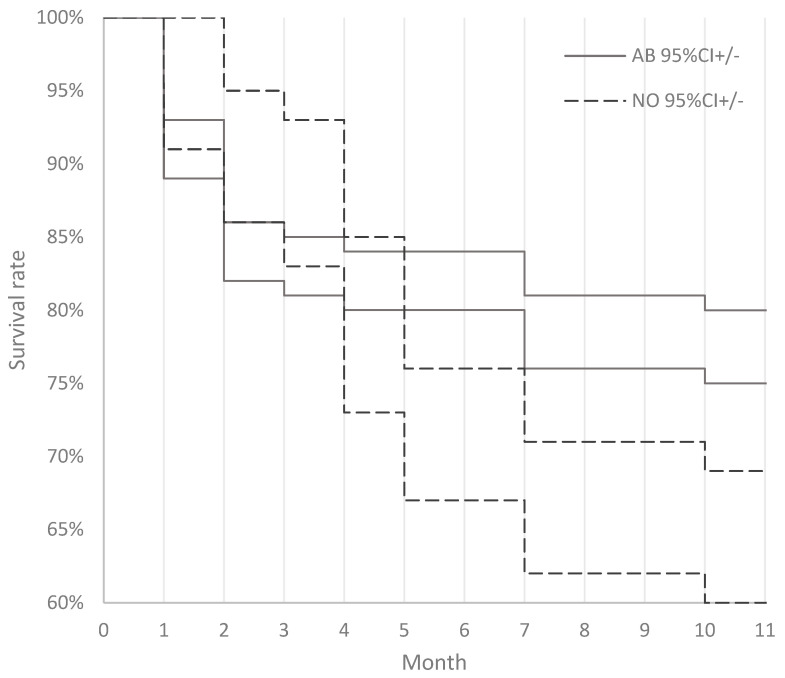
Survival analysis of cows included in the study for CM and culled for any reason during the 11 months of herd monitoring. The 95% CI for AB- (solid lines, n = 93) and NO-treated (dashed lines, n = 42) animals are shown. NS animals are not represented (n = 25).

By February 28, 2026, 45 of 174 cows (25.9%) were culled. In the AB group, the survival curve declined less rapidly (Figure 1) than in other groups. Culling causes were not reported by the farm staff in the cattle database. The final survival rate was 77.4% for the AB group (CI_95%_ [75.0; 80.0]) compared to 64.3% for the NO group (CI_95%_ [60.0; 69.0]).

The removal of animals from the herd within two months of the clinical event is a relevant indicator. Of the 45 animals removed, 22 (48.8%) were removed within 60 days of the CM, including seven within the following week. If we consider only the animals that could potentially have benefited from antibiotic treatment, the probability of removal from the herd within 60 days was 9.7%, 10.8%, and 17.6% for animals that received the AB, NO, and NS treatment options from farm staff, respectively (Table 6). The difference is not significant.

### 2.6. Between-Herd Variations

Significant variations existed between herds. The incidence rate of treated CM, as reported to the cattle database, varied from four to 36 cases per herd; the risk of antibiotic treatment ranged from RR = 0.143 (CI_95%_ [0.039; 0.523]) to RR = 1.429 (CI_95%_ [1.061; 1.923]). Only one herd showed improved health status (OR = 0.333, n.s.), while the other 10 showed a decline, with the worst case showing OR = 19.783 (CI_95%_ [1.014; 386.050], *p* < 0.05).

## 3. Discussion

Clinical mastitis is a common condition in dairy production. The clinical picture evolves very rapidly, with a more or less complete resolution of symptoms, sometimes accompanied by the elimination of the responsible bacterial agent. It is accepted that the administration of antibiotics, either by the intramammary route or systemically, can help limit the risk of pathogen persistence and thus prevent the deterioration of udder health. However, the value of antimicrobial therapy is greatest for pathogens that have a low rate of spontaneous cure and a high rate of treatment cure [12]. It is also accepted that obtaining information on the nature of the pathogen involved could improve disease management. Symptomatic treatment without knowledge of etiology results in unnecessary antimicrobial treatments (such as the use of antimicrobials for the treatment of culture-*negative* cases), and it is impossible to determine etiology without the use of diagnostic tests [12]. However, the time required to obtain this information is generally incompatible with the acute course of the disease. Under Danish strict regulation, farmers give a treatment that may or may not include antibiotics, but which they know generally has a favorable outcome on the clinical cases on their farm. Our study showed that this strategy is not satisfactory. Indeed, it reduced the amount of antibiotics used since only six out of 10 cows with clinical mastitis (58.1%) received AB treatment compared to the traditional blanket treatment. This aligns with previously published results [13,14,15,16,17]. But, overall, the probability ratio of giving AB when AB was deemed necessary was only 0.75, and more cows would probably have benefited from an antibiotic treatment. Also, one in two cows showed persistently high SCCs in the two months following the clinical event and its treatment, whatever treatment protocol was applied.

This study, however, has several limitations due to its design. Firstly, no selection was made of the animals enrolled in the study. Secondly, no guidelines were given to farm staff, who remained free to choose the treatment within the limits imposed by Danish regulations. Finally, the health status of the udder quarters was assessed using the resources available on the farm (SCC), but in the absence of microbiological testing after CM, this assessment remains imprecise.

The administrative requirement of collecting and analyzing a milk sample offered the opportunity to gather additional information that can help in making a better decision. In a study previously carried out in Denmark [18], “legislation” was the most significant driver for taking milk samples. Indeed, a microbiological examination performed at a veterinary clinic should allow for antibiotic use to be fairly close to what is expected, although slightly lower.

The quantitative results of milk cultures were quite similar between practicing veterinarians and a professional testing laboratory. The most frequent infectious causes were due to environmental streptococci (41.8%), primarily *Streptococcus uberis*, and coliform bacteria (14.4%), mainly *Escherichia coli*, followed by NASM organisms (10.6%). This situation is now commonly observed in Europe [16,17,19,20,21] and in other developed countries [8]. However, this distribution contrasts with the results of other studies on two points. Firstly, the number of negative results was rather low (<10%), whether with fresh or frozen milk samples. However, a study conducted in Denmark under comparable conditions in 2022 [22] reached the same conclusion. On the other hand, the low numbers of *Staphylococcus aureus* and non-aureus staphylococci infections are surprising. A recent meta-analysis [23] shows that the European region is dominated by staphylococcal infections (23%), followed by streptococci (12%) and then coliforms (10%). *S. aureus* infections generally predominate in Scandinavian countries. In the previously cited Danish study [22], *S. aureus* is the dominant species, and the total *S. aureus* plus non-aureus staphylococci far exceeds all other species.

The culture of a sample of mastitis milk, possibly after enrichment, followed by colony identification using MALDI-TOF technology, has become the gold standard in milk bacteriology. The concordance between results provided by professional laboratories and those obtained by veterinarians is questionable. Most often, veterinarians carry out Gram-staining or a potassium hydroxide test, a catalase test, a coagulase test, microscopy and phenotypical characterization based on both blood agar and CHROMagar^TM^ [22]. Such analyses result in a limited set of biochemical characteristics and do not appropriately differentiate the variety of species that can be present in milk and will grow on blood agar. Hence, despite being laborious, such analyses only allow for tentative identification of a limited range of bacteria—mainly the major pathogens [22]. This method lacks both sensitivity and specificity. In our study, not only were veterinarians not able to properly identify all the causative species of CM, but they get some species mixed up. At the clinic, veterinarians were not able to make NASM out, which is understandable. Unfortunately, they were unable to identify critical species such as *Streptococcus agalactiae* (only 2/5 identifications), and they found 10 *S. aureus* infections where only seven existed. Contaminated samples were poorly identified. When milk culture results are grouped in nine broad categories that make sense from a therapeutic point of view, outcomes from the professional and in-clinic laboratories fitted quite well.

Conceptually, the dairy farmer can be viewed as a mobile diagnostic system: highly adaptable, equipped with natural intelligence, and capable of storing complex animal histories in a biological “hard disk drive.” From an implementation standpoint, the marginal cost of utilizing the farmer test to guide treatment decisions is virtually zero. As a smart test for discriminating between AB, NO, and NS protocols, the farmer offers a unique and intriguing set of diagnostic advantages. However, like most tests developed to facilitate CM treatment decisions, the human test does not meet certain aspects of the ASSURED criterion (Accurate, Sensible, Specific, User-friendly, Rapid/Robust, Equipment-free and Deliverable) [24,25].

Within the cattle database, producers recorded three primary treatment decisions: NO (no intervention), NS (administration of an NSAID), and AB (administration of antibiotics). To align producer reports with available laboratory data, we assigned a standardized treatment protocol to each of the nine broad pathogen categories previously identified. Under this framework, IMIs related to Gram-positive bacteria, as well as mixed or contaminated cultures, were designated for AB treatment. Cases involving Gram-negative bacteria were assigned NO, while all other instances received NSAID. While this categorization is subject to debate—particularly the use of antibiotics to control certain Gram-positive organisms like *Corynebacterium* and NASM—it follows methodologies proposed by several authors [14,15,16]. This approach allowed us to compare actual producer decisions against recommended treatments derived from laboratory results. Overall, only 58.1% of CM cases were treated with antibiotics, representing a significant reduction compared to traditional blanket therapy. The relative risk of staff selecting AB treatment when it was clinically indicated was 0.755; notably, however, three out of 11 farms used more antibiotics than necessary. Finally, when treatment decisions relied solely on a producer’s perception of the historical effectiveness [12,18], only 48.8% of cows received the treatment deemed appropriate by laboratory standards, making their decisions essentially equivalent to a coin flip. The farmer test is therefore a convenient test, but its statistical characteristics are poor. In our study, 43.9% of CMs that could benefit from antibiotic treatment did not receive it, and 64.9% of cows that should have received symptomatic treatment (or none) were ultimately treated with antibiotics.

The same analysis can be conducted with veterinarians, using the same treatment rule, and assuming that the veterinarians rely strictly on their own test results. The performance of the veterinarian test is significantly better than that of the farmer test, and 83.3% of cows could benefit from the seemingly most useful treatment. With their slightly less sensitive milk culture methods, veterinarians will leave 12.0% of the cows that warrant it without antibiotic treatment. Conversely, 16.7% of cases will receive antibiotics when it is not necessary. Furthermore, in-clinic milk cultures do not adequately meet the desired characteristics expressed by farmers in a Dutch survey [26]. In this study, farmers expressed a need for tests that provide information on which antibiotic to use, and they would prefer to receive this information within 12 h or less. In-clinic milk testing does not meet the ASSURED criterion.

Our assessment of udder health status (UHS) is based on information available in the Cattle Database and generated by the DHI service. We therefore used SCCs as a proxy, utilizing the results of the two consecutive test days before and after CM, compared to the threshold of 200,000 cells/mL, and combining them according to the rule explained above. The granularity of the criterion used to classify animals as healthy (HTY) or infected (INF) is rather coarse. Indeed, the time between the last DHI test before CM and the first after was variable. Furthermore, the lack of information about the actual infection status—information not routinely available—makes statistical validation of the criterion impossible. However, the information is easy to obtain and can be analyzed on the farm without technological support, and it seemed sufficient to provide an acceptable representation of the health status. The use of last (td − 1) and penultimate (td − 2) test day results before the identification of an IMI, at a threshold of 200,000 cells/mL, has already been evaluated by others [27] with a positive predictive value of ≥90%. Similarly, high SCC status (>150,000 cells/mL for primiparous, >250,000 cells/mL for multiparous cows) on td − 1 and td − 2 combined, before CM are predictive of risk of CM [28]. Finally, SCC tends to increase sharply in the 21 days preceding the clinical episode [29]. Stating that nearly one in three cows was probably already infected before CM and that one in two remains infected (absence of cytological cure) during the 6 to 10 weeks following CM is a conclusion that does not contradict previous work, regardless of the method used to detect IMI.

By design, UHS evolution criterion with three outcomes (unsuccessful, inconsequential, success) is also crude. However, such simplified algorithms are used by others [30]. Criteria for determination of the successful treatment of CM are often difficult to establish. Without post-CM microbiological analyses, which were not the focus of this study, it was difficult to distinguish the deleterious effects of the disease from the lack of effectiveness of the treatments. Overall, cows with CM were 2.31-fold more likely to have a UHS status INF after the clinical episode. The treatment administered had no influence on UHS evolution outcome. This situation may be due to two non-mutually exclusive factors. On the one hand, one-third of the animals probably had a positive IMI status at the time CM manifested. The age of the infection was unknown, and in our study, the medical history was limited to two check-ups (approximately two months). On the other hand, the capability of treatment to eliminate IMI was low. It is interesting to underline that cows free of IMI before CM (HTY) were slightly more likely to be of status HTY after the clinical episode, than cows of status INF (52.5 vs. 42.2%, OR = 1.511, n.s.). Others [31] observe that high SCC cows before CM had reduced bacteriological cure rates compared to low SCC cows.

The evolution of the inflammatory status of the udder (cow composite SCC) is not a fair indicator of the evolution of the actual infectious status. Furthermore, information is also lacking on the reasons for cow removal from the herd, particularly for cows culled shortly after the clinical episode. However, the criteria used in this study suggest that the clinical outcomes of the different treatment options were evenly poor. The apparent cure rate (HTY status after cow spaying) was lower than the generally accepted bacteriological cure rate (65%), but our results did not contradict previously published work [32]. On the one hand, only a few preparations based on certain antibiotics show a cure rate that is statistically different from no treatment. On the other hand, the bacteriological cure rate with a non-steroidal anti-inflammatory drug such as ketoprofen is lower than that obtained with an antibiotic, but this difference is not noticeable when each group of pathogens is examined individually [33].

Retention within the herd is difficult to interpret since we did not have the exact cause of culling. Culling is influenced by many factors, and dairy farmers were allowed to remove cows without a withholding period if they had not received antimicrobial therapy. The follow-up period was exceptionally long (11 months) in this study. The short term retention rate was similar to the rate observed by others [30]. The difference in the retention rates between AB- and NO-treated animals should be handled with caution since there is insufficient evidence to suggest that they are influenced by choices made about mastitis treatment [12].

## 4. Materials and Methods

### 4.1. Consent to Participate in the Study

As a purely observational study, no interventions, interviews, or questionnaires were conducted with farm or veterinary personnel, and routine practices remained unchanged. The accumulated data were not utilized to guide or alter animal treatment protocols. Prior to data collection, informed consent was obtained from all farmers for the use of their data in scientific analysis. Additional ethical approval for human subject research was deemed unnecessary.

### 4.2. Study Population

The study population consisted of 12 specialized dairy farms in Jutland, Denmark, recruited for convenience sampling after the farmers and supervising veterinary clinics agreed to participate in the evaluation of decision-making rules regarding the use of antibiotics for mastitis treatment. The dominant breed was Holstein, with a minority of Jersey cows. Under Danish law, all livestock farms must report certain technical and health information to a national platform. The farms were therefore considered relatively similar with respect to the study’s objective, and it was not necessary to refine the inclusion criteria.

This study benefited from the fact that in Denmark, the use of antibiotics for mastitis treatment is only permitted if farmers collect a milk sample, which is then sent to a diagnostic service [Executive Order on the use, dispensing and prescription of veterinary medicinal products for animals by veterinarians, BEK #1227 of 19 November 2019, Ministry of Food, Agriculture and Fisheries]. However, farmers do not wait for the results to initiate treatment; rather, the results allow for retrospective verification of whether the farmer’s choice was appropriate and inform the veterinarian about the epidemiological situation.

### 4.3. On-Line Farm Records

In accordance with Danish regulations, all herd health events, including diseases and treatments, must be recorded by the farmer in the Danish Cattle Database (data provided by SEGES P/S, Aarhus N, Denmark). Therefore, farmers reported the treatments administered, which, in the case of mastitis (code 11), were coded AB (antibiotic treatment, no details), NO (no treatment at all), or NS (non-steroidal anti-inflammatory) in our study, along with the date of occurrence. After the fieldwork phase, a retrospective farm record review was conducted using individual data potentially related to cases of clinical mastitis occurring during the study period: reported treatments, udder health status (UHS) before and after the clinical event (two DHI recordings), and culling date. UHS before and after the clinical event were interpreted jointly according to the rules presented in Table 7. A disease outcome status was defined (unsuccessful, inconsequential, success), as was a change in status during the CM episode (Table 8).

A CUL variable was introduced. It measured the difference in days between the occurrence of CM and the date of culling; the culling reason was not reported.

### 4.4. Milk Sampling

#### 4.4.1. Milk Sample Management

Farmers were asked to collect two milk samples from each case of clinical mastitis detected between 1 March and mid-July 2025. The staff were provided with disposable gloves and disinfectant disposable wipes (chlorhexidine digluconate, ethyl alcohol, and isopropyl alcohol, Germo S.p.A., Cormano, Italy) to clean the teat apex. A first sample was collected in a sterile dry 30 mL polypropylene tube and immediately refrigerated (+2 to +6 °C). A second sample was collected shortly afterward in a 30 mL tube containing 2 mL of glycerol. After shaking, this sample was frozen (−18 °C) on the farm. All samples were identified and linked to the identity of the affected animal by cow’s individual ID; the date and affected quarter were recorded. Refrigerated samples were collected once or twice a week by the local veterinary clinic for microbiological analysis. The frozen samples were collected in two rounds and sent under negative temperature conditions to the analysis laboratory (LABEO, Saint-Lô, France) via express transport.

#### 4.4.2. Determining the Number of CM Cases to Be Sampled

The number of quarters to be sampled was estimated as follows. Based on a recent study conducted in Germany [19,33], Denmark [22] and The Netherlands [31], the ratio of situations where the use of antibiotics is advisable to those where antibiotics are not is estimated to be close to 70/30. If the treatment (AB or NO/NS) was assigned randomly, the probability that the farmer would choose one or the other would be 0.5. It was assumed that the piece of information, whatever its nature (farmer experience, microbiological examination, or result of any other testing device), would deflect the decision by 20 points in one direction or the other. Therefore, to observe this difference at the type I risk threshold α = 0.05 and for a power of 1 − β = 0.80, it was necessary to sample n = 2 × 94 = 188 quarters. The study designers thus aimed to collect milk samples from 200 cases of CM.

### 4.5. IMI Diagnostic Methods

#### 4.5.1. Farm Staff

Overall, the person responsible for milking and making decisions regarding clinical mastitis was considered a diagnostic tool alongside the cow. Farm staff based their decisions on their experience, farm routines, animal knowledge, and numerous subtle signals not investigated here. Their actions resulted in a decision with three outputs: AB (antibiotic treatment), NO (no treatment), or NS (non-steroidal anti-inflammatory drug). This decision was reported in the cattle database as previously mentioned.

#### 4.5.2. The Veterinary Clinic In-House Laboratory

The veterinary clinic laboratory, contracted to monitor herd health, received milk samples and cultured them on selective solid media in accordance with the recommendations of the NMC Laboratory Handbook on Bovine Mastitis (2017) [34] or acceptable derivative methods. This approach enabled the identification of contaminated samples, major bacterial pathogens, and certain bacterial groups within approximately 24 h.

The veterinary clinic results were ordered into eight coded categories to facilitate analysis: NEG (absence of growth of any bacteria), CONT (contaminated sample, when ≥3 phenotypically distinct colonies are detected), MIX (presence of several distinct colony types, up to a maximum of three), SAU (typical *Staphylococcus aureus* colonies), NASM (non-aureus staphylococci, coagulase-negative, and mammaliicocci), SUB (typical *Streptococcus uberis* colonies), OGP (other Gram-positive bacteria, including identifiable streptococci (*Str. agalactiae*, *Str. dysgalactiae*), streptococci-like organisms, and miscellaneous Gram-positive organisms. The list continued with COL (coliform bacteria such as *Escherichia coli* and *Klebsiella pneumoniae*) and finally OGN (unclassifiable and sparse Gram-*negative* bacteria).

#### 4.5.3. The Professional Microbiology Laboratory

The testing laboratory (LABEO Manche, Saint-Lô, France) fulfilled the requirements of the French NF EN ISO/IEC 17025:2017 standard [35] for performing these analyses. It was the gold standard of this study. The analytical technique is not described in detail but complied with the national guidelines (CNEVA, standard BA 140/00) [36]. In order to increase the sensitivity of the milk culture, a soy-trypicase broth (9 mL) was inoculated with 1 mL of the sample. On the other end, 10 µL of milk was plated onto fresh blood Colombia agar. If the culture did not grow on the agar after 24 h, a new blood agar was inoculated with 50 µL of the incubated broth. Petri dishes were read after 18, 24, 36 and 72 h incubation at 37 ± 1 °C, which made possible detection of bacteria at concentrations as low as 100 CFU/mL. Bacterial identification was performed by time-of-flight mass spectrometry. Matrix-Assisted Laser Desorption/Ionization Time-of-Flight (MALDI-TOF, Bruker Corporation, Karlsruhe, Germany) is a highly efficient mass spectrometry technique used to identify and analyze biomolecules, such as proteins, peptides, and polymers, by measuring their mass. It is widely used in clinical microbiology laboratories to identify bacteria and fungi in minutes rather than days.

The results were much more detailed than those from the veterinary clinic, but no MIC antibiotic sensitivity testing was performed. The results were categorized in the same way as described in the previous paragraph, and virtually the same decision rule was applied to the results during the analysis of results.

### 4.6. CM Treatment Strategy

Based on this categorization, a treatment rule was proposed (Table 9) which served as the basis for the analysis of the results.

### 4.7. Statistical Analysis

The statistical analysis of the data primarily involved comparing categorical variables. The tests used were the chi-square test to determine if there was an association between categorical variables, the z-test to determine whether a sample proportion differed from a reference population proportion, the calculation of the relative risk (RR), the odds ratio (OR) to compare proportions, and one-way ANOVA to test whether three or more group means differ. Statistical significance was assumed at α = 0.05. The data were collected and analyzed using Microsoft Office Excel 2013 (Microsoft Corporation, Redmond, WA, USA) and the online calculator 2026 Statistics.tools (https://statistics.tools/, accessed on 22 March 2026). The CM case of an udder quarter represented the statistical unit.

## 5. Conclusions

The Danish regulatory framework demonstrates that while administrative constraints can successfully decrease antimicrobial consumption for clinical mastitis therapy, such reductions do not inherently guarantee the judicious allocation of therapeutic resources. Without a comprehensive evaluation of individual animal health status, the full consequences of this approach remain difficult to quantify. To optimize the clinical management of CM, a collaborative diagnostic framework involving three main actors is proposed, and deserves to be tested in other work, on a larger scale and over longer periods. First, the producer must utilize rapid, quarter-level milk diagnostics to determine whether the specific case warrants immediate antimicrobial intervention. Second, the attending veterinarian should be responsible for monitoring shifting epidemiological patterns within the herd through the implementation of a statistically rigorous sampling protocol. Finally, specialized reference laboratories are essential to provide high-resolution data regarding microbial species identification and antimicrobial susceptibility profiles.

## Figures and Tables

**Table 1:** Comparison of the distribution of broad culture categories observed in the veterinary clinics (VETS) and the analysis laboratory (LAB), and respective advisable treatment.

	CONT/MIX	G+	NEG	G−
LAB (n = 174)	28	106	12	28
VETS (n = 160)	20	85	13	26
Desirable treatment	AB	AB	NO	NS

CONT/MIX: contaminated or mixed culture; G+: Gram-positive organism; NEG: negative culture; G−: Gram-negative organism. AB: antibiotic, NO: no treatment, NS: NSAIDs.

**Table 2:** Number of pathogen category-specific-related treatment and proportion of cases that received antimicrobials.

	Pathogen Category								
	CONT	MIX	SAU	NASM	SUB	OGP	NEG	COL	OGN
AB (n = 93)	9	6	4	10	29	14	4	14	3
NO (n = 42)	6	1	2	3	16	9	1	4	0
NS (n = 25)	1	2	1	3	8	2	3	3	2
%AB	56.3	66.7	57.1	62.5	54.7	56.0	50.0	66.7	60.0

COL: coliform organisms; CONT: contaminated culture; MIX: mixed culture; NASM: non-aureus staphylococci and mammaliicocci; NEG: negative culture; OGN: other Gram-negative organism; OGP: other Gram-positive organisms; SAU: *S. aureus*; SUB: *Str. uberis*. AB: antibiotic, NO: no treatment, NS: NSAIDs; %AB: percent of the category treated with ABs.

**Table 3:** Contingency table of treatments actually administered by the farm staff (FARMS) and potential recommendations based on laboratory results (LAB). NO = no treatment, NS = NSAIDs, AB = antibiotic.

		LAB			
		NO	NS	AB	
FARMS	NO	6	2	34	42
	NS	2	3	20	25
	AB	17	7	69	93
		25	12	123	160

The sum of each row or each column is shown in the last column or last row, respectively.

**Table 4:** Contingency table of potential veterinary prescriptions based on in-clinic milk cultures (VETS) and potential recommendations based on laboratory results (LAB). NO = no treatment, NS = NSAIDs, AB = antibiotic.

		LAB			
		NO	NS	AB	
VETS	NO	18	0	8	26
	NS	5	7	5	17
	AB	2	4	95	101
		25	11	108	144

The sum of each row or each column is shown in the last column or last row, respectively.

**Table 5:** Contingency table of CM treatment actually applied to animals and udder health status change. Unsuccessful = INF or HTY before CM then INF after; inconsequential = HTY before and after CM; success = INF before CM and HTY after treatment. NO = no treatment, NS = NSAIDs, AB = antibiotic.

		Udder Health Status Shift			
		Unsuccessful	Inconsequential	Success	
	NO	24	14	3	41
Treatment	NS	11	9	4	24
	AB	39	30	12	81
		74	53	19	146

The sum of each row or each column is shown in the last column or last row, respectively.

**Table 6:** Contingency table of cows withdrawn from the herd within 2 months after a CM episode in the different treatment groups, among cows that could potentially benefit from AB treatment.

		Treatment Actually Applied			
		AB	NO	NS	
Status	Culled	7	4	3	14
	Retained (>2 months)	65	33	14	112
		72	37	17	126

The sum of each row or each column is shown in the last column or last row, respectively. AB: antibiotic, NO: no treatment, NS: NSAIDs.

**Table 7:** Udder health status of animals subject to clinical mastitis over the study period. Based on individual SCC test days, penultimate (td − 2) and last (td − 1) controls prior to the clinical mastitis case, and first (td + 1) and second (td + 2) controls after.

Combined Test Day SCCs *	UHS
td − 2 > 199 et td − 1 > 199	INF
td − 1 > 199 et td − 1 > td − 2	INF
td + 1 > 199 et td + 2 > 199	INF
td + 2 > 199 et td + 2 > td + 1	INF
Other combinations	HTY

* SCC × 1000 cells/mL. UHS: udder health status; td: test day; SCC: somatic cell count; INF: infected; HTY: healthy.

**Table 8:** Udder health status (UHS) evolution through the clinical event (CM). (INF: infected; HTY: healthy).

UHS Before CM	UHS After CM	Evolution
INF	INF	Unsuccessful
HTY	INF	Unsuccessful
HTY	HTY	Inconsequential
INF	HTY	Success

UHS: udder health status; CM: clinical mastitis; INF: Infected; HTY: Healthy.

**Table 9:** Decision rule for the selection of treatment based on the category of the germ responsible.

Category	Therapeutic Decision
CONT, MIX, NASM, OGP, SAU, SUB	AB
COL, OGN	NO
NEG	NS

COL: coliform organisms; CONT: contaminated culture; MIX: mixed culture; NASM: non-aureus staphylococci and mammaliicocci; NEG: negative culture; OGN: other Gram-negative organism; OGP: other Gram-positive organisms; SAU: *S. aureus*; SUB: *Str. uberis*. ABs: antibiotic, NO: no treatment, NS: NSAIDs.

## Data Availability

The data from this study are available upon request to the corresponding author. They will be made available to the requester within a reasonable timeframe.

## Supplementary Materials

The following supporting information can be downloaded at https://www.mdpi.com/article/10.3390/antibiotics15060570/s1: Table S1. Species identified by the laboratory and veterinarians, and agreement between them. Figure S1. Categories of bacterial species identified by the professional laboratory and in-clinic milk testing services.

## Abbreviations

The following abbreviations are used in this manuscript:

CM	Clinical mastitis
DHI	Dairy herd improvement
IMI	Intramammary infection
SCC	Somatic cell count
NO	Absence of medicinal treatment
NS	Application of non-steroidal anti-inflammatory drug
AB	Application of antibiotic
