# Supplementary material for: Unsuitable Decisions and Persistent Infection: The Reality of Farmer and Veterinarian Mastitis Treatment Decisions in Denmark
Source: Antibiotics (Basel). 2026 Jun 3;15(6):570. doi: 10.3390/antibiotics15060570 (PMC13295821; doi:10.3390/antibiotics15060570)
Supplement: Supplementary file 1 [file antibiotics-15-00570-s001.zip › antibiotics-4315713-supplementary.pdf]

Supplementary Materials

Table S1. Species identified by the laboratory (LABO) and veterinarians (VETS), and agreement between them.

Species	LABO	VETS	% cultures identified by veterinarians that are also identified by the laboratory	% cultures identified by the Laboratory that are also identified by the veterinarians
Citrobacter koseri	1			0.0%
Escherichia coli	**17**	**15**	**66.7%**	**58.8%**
Klebsiella pneumoniae	**6**	**10**	**60.0%**	**100.0%**
Psychrobacter sanguinis	1			0.0%
Serratia spp	2			0.0%
*Pantoea agglomeran*s	1			0.0%
Enterobacter spp		1	0.0%	
Staphylococcus aureus	**7**	**10**	**30.0%**	**42.9%**
Staphylococcus spp.	1			*100.0%*
Staphylococcus epidermidis	2			*50.0%*
Staphylococcus haemolyticus	5			*80.0%*
Staphylococcus lentus	1			*0..0%*
Staphylococcus sciuri	2			*50.0%*
Staphylococcus simulans	4			*25.0%*
Staphylococcus succinus	1			*0.0%*
Staphylococcus xylosus	1			*0.0%*
**Non-aureus staphylococci**		**13**	**53..8%**	
Streptococcus agalactiae	**5**	**2**	**100.0%**	**40.0%**
Streptococcus dysgalactiae	10	7	71.4%	50.0%
Streptococcus uberis	**57**	**44**	**73.3%**	**57.1%**
Trueperella pyogenes	7	2	100.0%	28.6%
Corynebacterium bovis	2	1	0.0%	0..0%
Enterococcus saccharolyticus	1	1	0.0%	0.0%
Bacillus spp	1		0.0%	0.0%
Micrococcus		1	0.0%	
Candida spp		4	0..0%	
Mixed infection	10	14	14.3%	20.0%
No growth	12	13	30.8%	33.3%
**Contaminated**	**17**	**6**	**33.3%**	**11.8%**

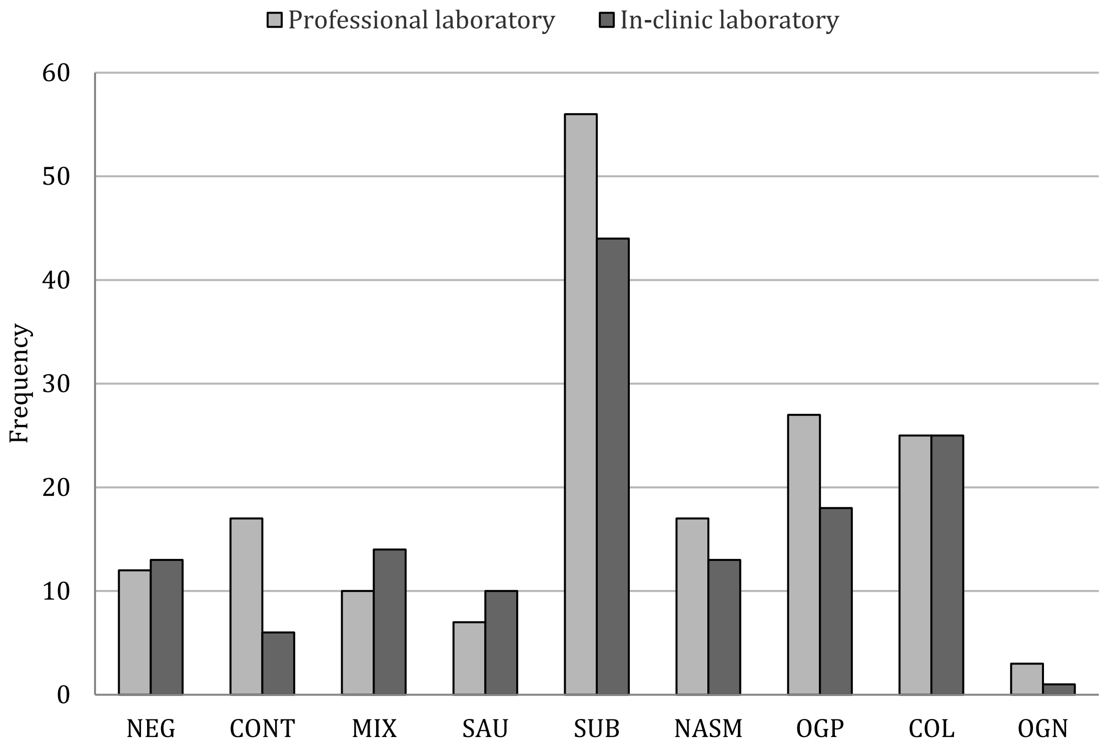

Figure S1. Categories of bacterial species identified by the professional laboratory and in-clinic milk testing services. COL: coliform organisms; CONT: contaminated culture; MIX: mixed culture; NASM: non-aureus staphylococci and mammaliicocci; NEG: negative culture; OGN: other Gram-negative organism; OGP: other Gram-positive organisms; SAU: *S. aureus*; SUB: *Str. uberis*.
